# Does chronic oral contraceptive use detrimentally affect C‐reactive protein or iron status for endurance‐trained women?

**DOI:** 10.14814/phy2.15777

**Published:** 2023-07-24

**Authors:** C. E. Badenhorst, A. D. Govus, T. Mündel

**Affiliations:** ^1^ School of Sport, Exercise and Nutrition Massey University Palmerston North New Zealand; ^2^ Discipline of Sport and Exercise Science La Trobe University Melbourne Victoria Australia; ^3^ Department of Kinesiology Brock University St. Catharines Canada

**Keywords:** cardiovascular risk, exercise, females, inflammation, ovarian hormones

## Abstract

**Purpose:**

Chronic use of the oral contraceptive pill (OCP) is reported to increase C‐reactive protein (CRP) levels and increase the risk of cardiovascular disease in premenopausal females.

**Methods:**

A secondary analysis of data from two research studies in eumenorrheic (*n* = 8) and OCP (*n* = 8) female athletes. Basal CRP and iron parameters were included in the analysis. Sample collection occurred following a standardized exercise and nutritional control for 24 h. Eumenorrheic females were tested in the early‐follicular and mid‐luteal phases, and the OCP users were tested in *quasi‐*follicular and *quasi‐*luteal phases (both active pill periods).

**Results:**

A main effect for group (*p* < 0.01) indicated that average CRP concentration was higher in OCP users compared with eumenorrheic females, regardless of the day of measurement within the cycle. Results demonstrate a degree of iron parameters moderation throughout the menstrual cycle that is influenced by basal CRP levels; however, no linear relationship with CRP, serum iron, and ferritin was observed.

**Conclusions:**

Basal CRP values were consistently higher in the OCP group despite participants being in a rested state. These results may indicate a potential risk of cardiovascular disease in prolonged users of the OCP when compared to eumenorrheic female athletes.

## 1 INTRODUCTION

C‐reactive protein (CRP) is a frequently measured indicator of long‐term inflammation (Cauci et al., 2017; Ridker et al., 2000). Within females, basal CRP levels greater than 1 mg L^−1^ are suggested to increase the risk of endothelial damage, myocardial infarction, thromboembolic events, and atrial fibrillation and have been used as a clinical threshold to determine cardiovascular disease risk (Cauci et al., 2017). Over the last decade, research in elite athletes (Larsen et al., 2020) and healthy recreationally active (Fedewa et al., 2017; Hinton et al., 2006; Quinn et al., 2021) female cohorts have consistently demonstrated an intermediate‐to‐high risk of cardiovascular disease (CRP >1 mg L^−1^) with long‐term oral contraceptive pill (OCP) use. Resting baseline CRP values in OCP users are consistently reported to be up to fourfold higher compared with naturally menstruating females, regardless of the time point of the sample collection (e.g., inactive/pill‐free days, mid‐active pill, or late pill days; Cauci et al., 2008; Quinn et al., 2021). Therefore, despite the wealth of research demonstrating the cardioprotective and anti‐inflammatory effects of regular exercise, the physiological benefits of exercise would appear to be attenuated by long‐term OCP use in premenopausal females. Approximately 33%–50% of athletic females use some form of hormonal contraception, with the majority of hormonal contraceptive users suggested to be using an OCP (Clarke et al., 2021; Martin et al., 2018; Nolan et al., 2023). While recent research has reported trivial effects of OCP use on the reliability and performance of exercise in athletic females (Elliott‐Sale et al., 2020; Zheng et al., 2021), the effect of OCP's on females' long‐term health is an ongoing area of research.

Medical use of the OCP is beneficial for various reproductive conditions and menstrual symptoms, including heavy menstrual bleeding and dysmenorrhea (Panicker et al., 2014). The impact of the OCP on menstrual bleeding, both volume of fluid lost and duration of bleeding, has been associated with a reduced risk of iron deficiency, which is a beneficial health outcome for athletic females who are susceptible to iron depletion (Fischer et al., 2021). Research that has compared athletic females' iron status between naturally menstruating and OCP users has reported lower transferrin saturation, serum iron, transferrin, and CRP levels in the mid‐late follicular and mid‐luteal phases in naturally menstruating females when compared to females during their active pill phase (e.g., ingesting standard doses of synthetic reproductive hormones). However, no differences in ferritin and hepcidin levels were reported between naturally menstruating and OCP users in this study (Alfaro‐Magallanes et al., 2022). Similarly, basal hepcidin levels have been reported to be consistent between pill phases (e.g., comparison between hormone‐free days and active pill phases; Sim et al., 2017); however, serum ferritin levels were reported to be higher in OCP users during the active pill phase (Sim et al., 2017). Therefore, variations in iron regulation for both naturally menstruating females and OCP users appear to be primarily determined by the individual's iron status. It is worth noting that most of the existing research on athletic and active females has primarily focused on differences in iron deficiency risk between OCP users and naturally menstruating females. To date, few studies have considered the impact of long‐term OCP use on inflammation, iron status, and subsequently cardiovascular risk in premenopausal females.

Therefore, the aim of this report was to compare the basal CRP and iron status of naturally menstruating and OCP users in a cohort of endurance‐trained athletic females. It is hypothesized that biomarkers of cardiovascular risk (CRP levels), and iron status, will be elevated in OCP users compared with naturally menstruating athletic females.

## 2 METHODS

This is a secondary analysis of data that was extracted from two studies that were completed at Massey University, Palmerston North, New Zealand. The methods for both experimental trials that are relevant to this paper will be presented in this section; however, full details of the methods have been published by Lei et al. (2017, 2019)). Both studies were approved by Massey University Human Ethics Committee: Southern A (14/99).

### 2.1 Participants

The data that are used for this analysis are from eight naturally menstruating females and eight females who were currently and had habitually used the combined monophasic OCP for more than 12 months. All participants provided informed consent. The monophasic OCP provides a constant dose of exogenous hormones for 21 days followed by 7 placebo pill days, with doses of ethinylestradiol of 20–35 μg, and progestins, specifically cyproterone acetate, levonorgestrel, and norethisterone, ranging from 0.1 to 2 mg. The descriptive characteristics of the participants are presented in Table 1. Females in both groups were well‐trained female cyclists and triathletes meeting the classification of Tier 3 athletes (McKay et al., 2022). Eumenorrheic naturally menstruating females were older and as such had more training years accumulated compared with the younger OCP female cohort. All other descriptive characteristics were not significantly different between groups.

**TABLE 1:** Mean ± standard deviation participant characteristics for eumenorrheic naturally menstruating women (natural) and combined oral contraceptive pill users (OCP).

Participant cohort	Age (years)	Height (cm)	Body mass (kg)	Body fat (%)	Absolute $\dot{V}$O_2max_ (L/min)	Relative $\dot{V}$O_2max_ (mL/kg/min)	Absolute peak power output (W)	Relative peak power output (W)	Training history (years)
Natural (*n* = 8)	35 ± 8	166 ± 9	64 ± 7	23 ± 6	3.1 ± 0.5	48 ± 7	282 ± 52	4.4 ± 0.8	8 ± 4
OCP (*n* = 8)	26 ± 7	167 ± 5	68 ± 9	25 ± 5	3.6 ± 0.6	53 ± 9	271 ± 19	4.0 ± 0.5	4 ± 3

### 2.2 Experimental overview

The data have been extracted from the baseline measurements of two projects, one focused on naturally menstruating females and a second on females using the combined monophasic OCP. During both projects, participants attended four experimental trials, two in each menstrual phase (early follicular: EF and mid‐luteal: ML) or OCP phase (*quasi*‐follicular: *q*F, and *quasi*‐luteal: *q*L) separated by 3 days (see Menstrual Cycle and OCP Phase below). Experimental trials were conducted at the same time of day (±1 h) and following >24 h of dietary and exercise control.

### 2.3 Preliminary testing

Graded exercise tests were undertaken in the *q*F (OCP group) or follicular (eumenorrheic group) phase to minimize the potential physiological effects of the menstrual/OCP cycle on physiological or functional capacity (e.g., $\dot{V}$O_2max_; Barba‐Moreno et al., 2022; Elliott‐Sale et al., 2020). Following body mass and height measurement, preliminary testing was conducted in a temperate laboratory environment (18–22°C) with a fan‐generated airflow of 19 km h^−1^ facing participants. A graded exercise test was undertaken on a cycle ergometer with the work rate beginning at 100 W. The graded exercise test consisted of increments at 25 W min^−1^ until volitional fatigue, with expired gases collected continuously throughout the test (Lei et al., 2017, 2019).

#### 2.3.1 Dietary and exercise control

The day of and prior to any experimental trial was marked by abstinence from alcohol, exercise, and only habitual caffeine use (as abstinence would confound withdrawal effects). Participants were provided with a standardized dinner (2 x Watties Snack Meals, Heinz Watties, New Zealand: 1363 (247) kJ providing 53 (6) g carbohydrate, 12 (4) g protein, and 8 (0.3) g fat) the night preceding the trial and were asked to consume the same light meal (consisting of toast or cereal) between 2 and 4 h prior to visiting the laboratory for the trial. Fluid was encouraged, and a euhydrated state was further ensured by instructing the participants to drink 500 mL of water 2 h prior to each trial.

#### 2.3.2 Menstrual cycle and OCP phase

The eumenorrheic participants were tested during the EF and ML phases, to maximize differences in estrogen and progesterone concentrations. Testing occurred on days 3 (1) and 6 (1) (EF), and 18 (2) and 21 (3) (ML) following the start of menses, with 12 (2) days separating the second EF and first ML trials. A progesterone level of >5 ng mL^−1^ was used as evidence that ovulation had occurred (Leiva et al., 2015; Schaumberg et al., 2017).

Participants who were in the OCP group were tested during the *q*F and *q*L phases to permit comparison with the eumenorrheic group. Testing occurred on Days 3–5 and 18–20 following the *start of OCP use*. Therefore, the OCP users were tested on Days 10–12 (*quasi* mid‐late follicular) and 25–27 (*quasi* mid‐late luteal) following the *start of their withdrawal bleed*. These testing days for the OCP group were selected to ensure that the hormonal profile during data collection was analogous with the eumenorrheic group. The EF and QF were selected as both represent the lowest hormonal profile for both naturally menstruating females and OCP users. Testing for the OCP group was not completed in the “pill free” days, due to the potential increase in endogenous reproductive hormone levels that occurs when synthetic hormones are no longer present to suppress the activity of the hypothalamic–pituitary axis (Elliott‐Sale et al., 2020). The ML and QL phases were selected as both provide represent the highest concentration of endogenous and synthetic hormones in the naturally menstruating and OCP group, respectively. In addition, the timing of the QF and QL phase testing in OCP users would enable researchers to determine whether endogenous biphasic rhythms of the menstrual cycle on thermoregulatory responses were maintained during active OCP use, a result that has previously been presented in research, but required validation (Grucza et al., 1993; Lei et al., 2019; Sunderland & Nevill, 2003).

#### 2.3.3 Baseline testing protocols

On arrival to the laboratory participants voided to produce a urine sample to confirm a urine specific gravity <1.010 and hence euhydration. Nude mass was recorded, and a blood sample was obtained from the antecubital vein.

### 2.4 Measurements

#### 2.4.1 Anthropometric

Participant height and mass were measured using a stadiometer (Seca, Germany; accurate to 0.1 cm) and scale (Jadever, Taiwan; accurate to 0.01 kg), from which surface area was estimated (Dubois & Dubois, 1916). Body composition was measured using multifrequency bioelectrical impedance analysis (InBody 230, Korea) using a standard procedure (Kyle et al., 2004).

#### 2.4.2 Respiratory

Expired respiratory gases were collected and analyzed to calculate $\dot{V}$O_2_ and carbon dioxide elimination ($\dot{V}$CO_2_), ventilation ($\dot{V}$_E_), and respiratory exchange ratio (RER), using an online, breath‐by‐breath system (VacuMed Vista Turbofit) using a 30‐s average. The system was calibrated before each trial using β‐standard gas concentrations and a 3‐L syringe (VacuMed).

#### 2.4.3 Hormonal

Blood was collected by venepuncture into a vacutainer (Becton‐Dickinson, UK) containing a clot activator. Following inversion and clotting, the whole blood was centrifuged at 4°C and 805 *g* for 12 min and aliquots of serum were transferred into Eppendorf tubes (Genuine Axygen Quality) and stored at −80°C until further analysis. Serum samples were analyzed using enzyme‐linked immune assays for 17β‐oestradiol (E2) (Demeditec Diagnostics, Kiel, Germany) and progesterone (P4) (IBL International, Hamburg, Germany) with a sensitivity of 6.2 pg mL^−1^ and 0.045 ng mL^−1^, respectively, and an intra‐assay variation of <6% and <7%, respectively. Serum CRP concentrations were analyzed using immunoturbidimetric assays (Randox Australia Pty Ltd, Parramatta, Australia), with a sensitivity of 0.252 mg L^−1^ and an intra‐assay variation of <2%. Serum interleukin‐6 (IL‐6) concentrations were analyzed using enzyme‐linked immunosorbent assays (R&D Systems, Minneapolis, MN, USA), with a sensitivity of 0.04 pg mL^−1^ and an intra‐assay variation of <8%. Serum iron parameters (ferritin, transferrin, and iron) were determined using an automated analyzer (Cobas E601, Roche Diagnostics, Auckland, New Zealand) with the following sensitivity and repeatability: 0.5 μg L^−1^ and <4% (ferritin), 0.1 g L^−1^ and <3% (transferrin) 0.9 μmol L^−1^ and <2% (iron). The following calculations determined total iron binding capacity (TIBC) and transferrin saturation (TSAT):

TIBC=Transferrin×22.8

$$\text{TSAT}=\frac{\text{iron}\times 100\%}{\text{TIBC}}$$

### 2.5 Statistical analysis

Linear mixed models were fit using the *nlme* (Pinheiro et al., 2022) and generalized linear mixed model fit using the *lme4* package (Bates et al., 2015) in the R Statistical Computing language (R Core Team, 2022) to examine the difference in serum iron parameters (continuous variables: iron, ferritin, and transferrin), inflammatory cytokines (continuous variables: IL6, CRP), sex steroid hormones (continuous variables: E2, P4), and body core temperate between OCP and non‐OCP users. All models were fit with fixed effects for menstrual cycle day (categorical variable, 3 levels: Day 6, Day 18, and Day 21) and group (categorical variable, 2 levels: OCP users and non‐OCP users) and a random intercept per participant. Sex steroid hormones (E2 and/or P4) were included as continuous covariates in the iron parameter (iron, ferritin, and transferrin) and inflammatory cytokine models (CRP and IL‐6). Age was also included as a covariate in the CRP model. CRP was included as a continuous covariate in the E2, P4, and iron parameter models. Where appropriate, the residual (level 1) variance was allowed to vary between OCP and non‐OCP users over time, and temporal autocorrelation was modeled using an exponential covariance structure. Candidate nested models were compared using Bayesian Information Criteria (BIC) (Schwarz, 1978). Models with a lower BIC value were considered parsimonious (i.e., the simpler model was favored). Marginal (the variance explained by the fixed effect alone) and conditional *R* ^2^ values (the variance collectively explained by the fixed and random effects) were calculated as an absolute metric of model fit (Nakagawa & Schielzeth, 2013) and checked via visual inspection of the model quantile‐quantile (Q‐Q) plots using the resid_panel() function in the *ggResidpanel* package (Goode & Rey, 2022).

Visual inspection of Q‐Q plots determined that model residuals for E2, P4, IL‐6, CRP, ferritin, and iron did not follow a Gaussian distribution. Hence, these models were refit using a generalized linear mixed model with a Gamma distribution and log link function. Since E2 and P4 data included zero values, 0.01 (a value below the limit of detection for each assay) was added to all zero values to enable estimation. Q‐Q plots of the Gamma regression models indicated that residuals were approximately normally distributed. 95% profile confidence intervals were calculated to denote the imprecision of the model fixed effect parameter estimates.

## 3 RESULTS

Table 2 presents the median (range) sex hormone, inflammatory cytokines, and iron parameter concentrations for OCP and non‐OCP users over time.

**TABLE 2:** Median (range) values for sex hormones (estrogen and progesterone) inflammatory proteins (CRP and IL‐6) and iron parameters (D‐G) for OCP and non‐OCP users over time.

Group	Day	Iron (μmol L^−1^)	Ferritin (μg L^−1^)	Transferrin (g L^−1^)	IL‐6 (pg mL^−1^)	CRP (mg L^−1^)	E2 (pg mL^−1^)	P4 (ng mL^−1^)
		Median (range)	Median (range)	Median (range)	Median (range)	Median (range)	Median (range)	Median (range)
Natural	3	14.0 (6–25)	45.3 (26–92)	2.4 (2.2–3.0)	0.9 (0.7–1.3)	0.4 (0.2–1.0)	58.3 (37.0–205.9)	0.4 (0.2–1.3)
Natural	6	16.5 (8–26)	50.5 (20–99)	2.5 (2.3–3.1)	0.9 (0.6–1.5)	0.3 (0.1–0.9)	60.4 (40.5–255.5)	0.6 (0.2–1.1)
Natural	18	16.0 (11–32)	49.3 (26–92)	2.5 (2.0–3.1)	0.7 (0.3–1.1)	0.4 (0.1–1.1)	92.9 (56.4–297.3)	9.2 (5.8–23.6)
Natural	21	19.5 (11–34)	48.5 (35–88)	2.6 (2.2–3.1)	0.7 (0.6–1.0)	0.2 (0.1–0.9)	82.1 (64.9–354.9)	14.2 (9.1–24.9)
OCP	3	14.9 (8–33)	46.0 (8–121)	2.9 (2.5–3.9)	1.1 (0.3–2.7)	1.8 (0.4–4.5)	13.3 (0.2–79.2)	0.1 (0.0–0.4)
OCP	6	14.0 (6–29)	38.0 (9–87)	3.1 (2.5–3.7)	1.1 (0.5–3.2)	1.5 (0.7–6.8)	18.3 (0.0–89.4)	0.2 (0.1–0.5)
OCP	18	16.5 (6–26)	46.6 (5–76)	3.2 (2.7–4.0)	1.9 (0.3–2.7)	1.9 (0.6–7.5)	16.4 (0.6–74.8)	0.2 (0–0.4)
OCP	21	14.5 (10–20)	42.8 (8–79)	3.2 (2.5–4.2)	1.0 (0.5–4.8)	1.7 (0.1–5.1)	14.7 (1.9–101.8)	0.2 (0.1–0.5)

Table 3 presents the parsimonious model (lowest BIC value) for each blood parameter. Results for all models, including their model fit statistics, diagnostic plots, and R code to reproduce the statistical analysis are available at the following link (https://github.com/AndyGovus23/Badenhorst‐et‐al.‐2023‐_Iron_Sex_Hormones).

**TABLE 3:** Parsimonious models for all variables. Transferrin is presented on the response (raw unit) scale, with all other variables reported on the log scale.

Variables	E2			P4			CRP			IL6			FE			FER			TRANS		
	Estimates	CI	*p*	Estimates	CI	*p*	Estimates	CI	*p*	Estimates	CI	*p*	Estimates	CI	*p*	Estimates	CI	*p*	Estimates	CI	*p*
(Intercept)	29.8	16.86–52.68	<0.001	0.64	0.49–0.84	0.002	0.79	0.53–1.16	0.224	1.01	0.78–1.30	0.953	15.34	12.33–19.07	<0.001	40.23	27.20–59.51	<0.001	2.84	2.66–3.02	<0.001
Day (6)	0.85	0.69–1.05	0.127	0.40	0.32–0.51	<0.001	1.13	0.96–1.33	0.129	1.08	0.91–1.28	0.395	0.96	0.87–1.05	0.365	1.04	0.95–1.14	0.369	−0.09	−0.16 – −0.01	0.021
Day (18)	0.88	0.71–1.08	0.202	0.49	0.38–0.62	<0.001	0.97	0.83–1.14	0.726	1.02	0.85–1.21	0.858	0.94	0.86–1.04	0.220	0.91	0.83–0.99	0.030	−0.02	−0.09 – 0.05	0.551
Day (21)	1.12	0.92–1.38	0.259	1.96	1.53–2.49	<0.001	1.10	0.93–1.29	0.267	0.95	0.80–1.14	0.587	1.03	0.94–1.14	0.520	1.02	0.93–1.11	0.685	0.05	−0.02 – 0.12	0.171
Group (OCP)	2.78	1.57–4.92	0.001	4.04	3.10–5.26	<0.001	0.47	0.32–0.70	<0.001	0.76	0.59–0.98	0.037	1.05	0.84–1.30	0.680	1.21	0.82–1.78	0.340	−0.29	−0.47 – −0.11	0.002
Day (6) * Group (OCP)	0.97	0.79–1.20	0.790	0.47	0.38–0.60	<0.001	1.17	1.00–1.38	0.054	1.13	0.95–1.34	0.167	0.90	0.82–1.00	0.041	0.90	0.83–0.98	0.022	0.03	−0.04 – 0.10	0.426
Day (18) * Group (OCP)	0.93	0.76–1.14	0.471	0.47	0.37–0.60	<0.001	0.97	0.82–1.14	0.679	1.10	0.93–1.31	0.263	1.01	0.91–1.11	0.905	1.04	0.96–1.14	0.328	0.04	−0.04 – 0.11	0.325
Day (21) * Group (OCP)	1.09	0.89–1.34	0.391	2.04	1.60–2.60	<0.001	1.00	0.85–1.17	0.955	0.81	0.68–0.96	0.017	1.03	0.94–1.14	0.490	1.02	0.93–1.11	0.729	−0.05	−0.12 – 0.02	0.167
Random Effects																					
σ^2^	0.19			0.26			0.18			0.19			0.07			0.07			0.03		
τ_00_	0.31 _ID_			0.09 _ID_			0.19 _ID_			0.09 _ID_			0.05 _ID_			0.14 _ID_			0.12 _ID_		
ICC	0.62			0.25			0.52			0.33			0.45			0.66			0.82		
*N*	16 _ID_			16 _ID_			16 _ID_			16 _ID_			16 _ID_			16 _ID_			16 _ID_		
Observations	64			64			64			64			64			64			64		
Marginal *R* ^2^/Conditional *R* ^2^	0.685/0.881			0.903/0.927			0.617/0.816			0.246/0.492			0.068/0.485			0.165/0.720			0.376/0.886		

### 3.1 Sex steroid hormones

Controlling for CRP concentration (χ^2^ (1) = 0.02, *p* = 0.90) did not meaningfully improve the E2 model fit compared with the unadjusted model (BIC = 600.98 vs. 596.84). In the unadjusted E2 model, the results of the omnibus ANOVA test revealed no effect for time × group (χ^2^ (3) = 1.01, *p* = 0.80), no main effect for day (χ^2^ (3) = 6.03, *p* = 0.11) but a main effect for group (χ^2^ (1) = 12.98, *p* < 0.01). Fixed effect explained 69% of the total variance and fixed and random effects collectively explained 88% of the total variance.

Controlling for CRP concentration (χ^2^ (1) = 0.49, *p* = 0.49) did not improve model fit compared to the unadjusted P4 model (BIC = 98.42 vs. 94.72). In the unadjusted model, P4 concentration differed over time between OCP users vs. non‐OCP users (χ^2^ (3) = 116.20, *p* < 0.01), with a main effect for day (χ^2^ (3) = 142.85, *p* < 0.01) and group (χ^2^ (1) = 112.26, *p* < 0.01). Model fixed effects explained 90% of the total variance, with the fixed and random effects collectively explaining 93% of the total variance.

P4 concentration has been back transformed [exp(β‐1) × 100] into percentages to assist post hoc analysis interpretation. Post hoc analysis of the unadjusted model revealed that non‐OCP users had 267% (95% CI: [84, 632]), 263% (95% CI: [81, 624]), 6703% (95% CI: [3265, 13,600), and 7726% (95% CI: 3829, 15,502]) higher P4 concentrations compared with OCP users at Days 3, 6, 18, and 21, respectively.

### 3.2 Inflammatory cytokines

Controlling for E2 (χ^2^ (1) = 0.16, *p* = 0.69), P4 (χ^2^ (1) = 0.18, *p* = 0.67) concentrations and age (χ^2^ (1) = 0.27, *p* = 0.61) did not improve CRP model fit compared with the unadjusted model (BIC = 116.11 vs. 104.33). In the unadjusted CRP model, the results of the omnibus ANOVA test revealed no interaction effect for the day × group (χ^2^ (3) = 3.60, *p* = 0.31), no main effect for the day (χ^2^ (3) = 6.29, *p* = 0.10), and a main effect for group (χ^2^ (1) = 14.17, *p* < 0.01).

Controlling for E2 (χ^2^ (1) = 0.21, *p* = 0.64) and P4 concentrations did not improve model fit compared to the unadjusted IL6 model (BIC = 126.95 vs. 121.42). With our sample size, there was insufficient evidence to reject the null hypothesis that change in IL‐6 concentration between each menstrual cycle phase differed between OCP users vs. non‐OCP users (χ^2^ (3) = 7.03, *p* = 0.07). There was no main effect of day (χ^2^ (3) = 0.99, *p* = 0.80), but there was a main effect for group (χ^2^ (1) = 4.56, *p* = 0.03).

### 3.3 Iron parameters

Controlling for E2, P4, CRP, and IL6 did not improve model fit compared with the unadjusted model both for serum iron (BIC = 415.36 vs. 405.61) and ferritin (BIC = 544.16 vs. 531.70). In the unadjusted serum iron model, the omnibus ANOVA test indicated no day × group (χ^2^ (3) = 4.86; *p* = 0.18), day (χ^2^ (3) = 0.17; *p* = 0.68), or group effect (χ^2^ (1) = 4.56, *p* = 0.68). Serum ferritin results were similar, with the omnibus ANOVA test indicating no day x group (χ^2^ (3) = 5.76; *p* = 0.12), day (χ^2^ (3) 5.12; *p* = 0.16), or group effect (χ^2^ (1) = 0.93; *p* = 0.34) for serum ferritin. In the serum iron model, the fixed effects explained 7% of the total variance, with the fixed and random effects collectively explaining 49% of the total variance. Notably, with our sample size, controlling for CRP (χ^2^ (1) = 3.68; *p* = 0.06) did not improve the serum iron model fit compared with the unadjusted model (BIC = 406.18 vs. 405.16) but improved marginal *R* ^2^ to 17% and conditional *R* ^2^ to 54%. Similarly, while controlling for CRP concentration (χ^2^ (1) = 3.69; *p* = 0.05) did not improve serum ferritin model fit compared with the unadjusted model (BIC = 532.22 vs. 531.70) marginal *R* ^2^ and conditional *R* ^2^ values increased to 23% and 72%, respectively. While there is insufficient evidence to reject the null hypothesis of no linear relationship between serum iron or ferritin and CRP concentration with our current sample size, CRP may moderate serum iron and ferritin concentrations throughout the menstrual cycle.

Controlling for E2, P4, CRP, and IL‐6 did not improve model fit compared with the unadjusted serum transferrin model (BIC = 108.27 vs. 68.08). In the unadjusted serum transferrin model, the omnibus ANOVA test indicated no time × day effect (χ^2^ (3) = 2.80; *p* = 0.42), but a main effect for day (χ^2^ (3) = 7.89; *p* = 0.05) and group (χ^2^ (1) = 10.43; *p* < 0.01). Fixed effects explained 38% of the total variance and fixed and random effects collectively explained 89% of the total variance, respectively. Controlling for E2 (χ^2^ (1) = 4.32; *p* = 0.04) improved the marginal *R* ^2^ to 44% and the conditional *R* ^2^ to 89% but did not improve model fit compared with the unadjusted serum transferrin model (BIC = 79.82 vs. 68.08).

## 4 DISCUSSION

The main finding of this report is average basal CRP values in well‐trained endurance, and rested females (i.e., Tier 3 athletes) are higher in OCP users when compared to naturally menstruating females. This result remains consistent regardless of the day of measurement within the cycle. Second, there was no linear relationship with CRP and serum iron or ferritin; however, the results would suggest a degree of iron parameters moderation throughout the menstrual cycle that is affected by CRP levels.

The higher CRP levels in OCP users when compared to naturally menstruating females reported here are in line with most of the available research. Much of this previous research has been completed in sedentary or recreationally active females (Cauci et al., 2017; Larsen et al., 2018; Quinn et al., 2021), cohorts typically completing less exercise and may be considered to have lesser cardioprotective and anti‐inflammatory effects from their exercise volume. Subsequently, these individuals may be expected to present with elevated basal CRP values in comparison with well‐trained female athletes (Cauci et al., 2017). However, in Tier 1 and 2 elite female athletes (McKay et al., 2022), CRP levels in OCP users were reported to be ~2 mg L^−1^, approximately threefold higher than mean CRP levels in naturally menstruating females (0.3–0.5 mg L^−1^) of matched athletic ability (Larsen et al., 2020). In these elite athletes, no naturally menstruating females but nine OCP users were considered high risk for cardiovascular disease (CRP >3 mg L^−1^). Equal numbers of the OCP users and naturally menstruating elite females were considered to have an intermediate risk of cardiovascular disease (CRP 1–3 mg L^−1^), a result that may be due to the lack of standardization of the rested blood sample collection protocol. Samples were collected from elite female athletes throughout the day, with athletes either in a rested or post training recovery state (Larsen et al., 2020). Interestingly, in the absence of sample collection control, the number of naturally menstruating elite athletes that were considered to be at low risk of cardiovascular disease (CRP <1 mg L^−1^) was double that of the number of OCP users (Larsen et al., 2020). The results of the current study reported median CRP levels in OCP users (~2 mg L^−1^) and naturally menstruating females (~0.4 mg L^−1^), with one naturally menstruating female considered to have an intermediate risk of cardiovascular disease and the remaining (*n* = 7) presenting with CRP levels <1 mg L^−1^. Conversely, five OCP users in the current study were at an intermediate risk and two were at a high risk of cardiovascular disease. Previous research has suggested that CRP may increase substantially in late adolescence in females, a factor that was considered in this analysis with the OCP group being ~10 years younger than the EUM group (Shanahan et al., 2013). However, age had no effect on the CRP model, a result that may be due to a lack of differences in body mass and percentage of fat mass between the OCP and EUM groups (Shanahan et al., 2013). In addition, it is noted that within age matched late adolescence females the use of OCP's was found to substantially increase basal CRP levels (Shanahan et al., 2013). Therefore, the current study reaffirms that regardless of the rested physiological state or age of these well‐trained female athletes, the CRP levels in long‐term users of OCP are likely to be twofold higher than naturally menstruating females of equivalent training and anthropometric status. Thus, the current evidence in well‐trained and elite female athletes demonstrates that the negative association between CRP levels and exercise volume is absent in OCP users and the long‐term effect on the health of these female athletes may need to be considered in practice and future research.

The function of the OCP is to downregulate the hypothalamic‐pituitary‐ovarian axis and subsequently the endogenous levels of estrogen and progesterone (Elliott‐Sale et al., 2013). As a result, the circannual rhythm of the uterus in premenopausal females is suppressed, reducing the likelihood of pregnancy occurring. However, an ensuing effect is a reduction in the length and volume of menstrual blood loss, and in some instances the absence of menstrual bleeding in OCP users (Clancy, 2009; Milman et al., 1993). Inference from research has suggested that prolonged use of OCP is a beneficial intervention for preventing iron depletion and the risk of iron deficiency as a result of the observed changes to menstrual bleeding (Alfaro‐Magallanes et al., 2022). Within athletic females, it is worth noting that iron status is consistently challenged through increases in iron loss through exercise‐associated mechanisms, inflammatory derived increases in hepcidin and subsequent alterations in iron regulation, increased risk of heavy menstrual bleeding, and low dietary iron intake from various dietary intake behaviors and patterns (e.g., vegan, vegetarian, macronutrient restrictive, and dietary restrictive; Badenhorst et al., 2022). The reduction in total iron lost through one of these mechanisms is likely to be considered beneficial for the sustainability of female athletic performance. Regardless, the current study would suggest that iron parameters were not different between OCP users and naturally menstruating females, consistent with prior research that has shown no difference in iron deficiency risk between OCP users and nonusers (Casabellata et al., 2007). In contrast, research in recreationally active females has reported higher serum iron and transferrin levels throughout the OCP cycle with no differences in serum ferritin, hepcidin, hemoglobin, and IL‐6 between OCP users and naturally menstruating females (Alfaro‐Magallanes et al., 2022). Elevations in transferrin saturation and serum iron in OCP users have previously been suggested to be due to hepatic effects of exogenous estrogens (McKnight et al., 1980), with OCP users more likely to have transferrin saturation levels >45% and be considered at risk of hemochromatosis (McKnight et al., 1980). Thus brand, duration, and behavioral use of OCP may be contributing to the variations seen in serum iron and transferrin between studies in active females and may need to be considered in future research. Despite the differences in serum iron and transferrin saturation between the current and previous research, both studies have reported elevated CRP levels in OCP users, regardless of the phase of data collection (active vs withdrawal) when compared to naturally menstruating females (Alfaro‐Magallanes et al., 2022). The impact of elevated cardiovascular disease risk (i.e., elevated CRP levels) and increased risk of hemochromatosis that appears to be evident with prolonged use of synthetic reproductive hormones by premenopausal females is an area of health and exercise performance research that remains to be investigated.

Within the current study, the inflammatory cytokine IL‐6, an upregulator of hepcidin levels that is frequently measured in research that considers iron status in active individuals, was not different between OCP users and naturally menstruating females. Similar results have been reported in previous research (Alfaro‐Magallanes et al., 2022; Sim et al., 2017) prior to exercise. Glycogen availability is a modulator of IL‐6 levels (Steensberg et al., 2000), and in a well‐controlled and standardized intervention research trial, as per this study and previous research (Alfaro‐Magallanes et al., 2022; Sim et al., 2017), glycogen levels in skeletal muscle are not likely to be different between females regardless of OCP use or not. Therefore, we would not expect differences between groups regardless of the time of the cycle of data collection. Similarly, hepcidin levels between OCP users and naturally menstruating females have not been shown to be different (Alfaro‐Magallanes et al., 2022; Sim et al., 2017). This would suggest that regulation of iron status at rest in both OCP users and naturally menstruating females is likely to occur in response to changes in measured iron parameters and at rest is not influenced by inflammation or reproductive hormones, both exogenous and endogenous.

The novelty of the results is that they align with previous research in well‐trained athletes (Tier 3 or higher) despite the strict control of the rested and fed state prior to the blood sample collection. The current study provides evidence of variations in cardiovascular disease risk, as indicated by CRP levels, in well‐trained females, controlling for the rested state prior to sample collection. The results have not shown any variations in iron parameters between the OCP users and naturally menstruating females, a result that does align with previous research but in this study is likely due to the small sample size. Future research is required to determine the difference in CRP levels between OCP users and naturally menstruating females in addition to defining the variation in measured iron parameters throughout the menstrual cycle and OCP cycle. Such research may add to the current literature on the health status of females that consistently utilized the OCP. Future research may seek to determine the impact of chronically elevated CRP levels in OCP users on active females (both recreationally active and athletes) exercise performance, recovery, and health.

## 5 CONCLUSION

This study has provided complementary evidence to the current literature, of elevated CRP levels in females that have used OCP for >12 months. The OCP users in this study were classified at an intermediate and high risk for cardiovascular disease, with one only OCP user considered to be at low risk for cardiovascular disease.

## AUTHOR CONTRIBUTIONS

All authors contributed significantly to conceptualization and design, data analysis, interpretation and drafting of the article. All authors approved the final manuscript.

## CONFLICT OF INTEREST STATEMENT

There are no conflicts of interest to report.

## FUNDING INFORMATION

This study was supported by the New Zealand‐Japan Joint Research Project Programme, under Catalyst: Seeding funding from Royal Society Te Apārangi (funds held by Toby Mündel).
